# Animal Models of Diabetic Neuropathy: Progress Since 1960s

**DOI:** 10.1155/2013/149452

**Published:** 2013-07-29

**Authors:** Md. Shahidul Islam

**Affiliations:** Department of Biochemistry, School of Life Sciences, University of KwaZulu-Natal (Westville Campus), Durban 4000, South Africa

## Abstract

Diabetic or peripheral diabetic neuropathy (PDN) is one of the major complications among some other diabetic complications such as diabetic nephropathy, diabetic retinopathy, and diabetic cardiomyopathy. The use of animal models in the research of diabetes and diabetic complications is very common when rats and mice are most commonly used for many reasons. A numbers of animal models of diabetic and PDN have been developed in the last several decades such as streptozotocin-induced diabetic rat models, conventional or genetically modified or high-fat diet-fed C57BL/Ks (db/db) mice models, streptozotocin-induced C57BL6/J and ddY mice models, Chinese hamster neuropathic model, rhesus monkey PDN model, spontaneously diabetic WBN/Kob rat model, L-fucose-induced neropathic rat model, partial sciatic nerve ligated rat model, nonobese diabetic (NOD) mice model, spontaneously induced Ins2 Akita mice model, leptin-deficient (ob/ob) mice model, Otsuka Long-Evans Tokushima Fatty (OLETF) rat model, surgically-induced neuropathic model, and genetically modified Spontaneously Diabetic Torii (SDT) rat model, none of which are without limitations. An animal model of diabetic or PDN should mimic the all major pathogeneses of human diabetic neuropathy. Hence, this review comparatively evaluates the animal models of diabetic and PDN which are developed since 1960s with their advantages and disadvantages to help diabetic research groups in order to more accurately choose an appropriate model to meet their specific research objectives.

## 1. Introduction

The term “diabetes” was first coined by Araetus of Cappodocia (81-133AD). Later, the word “mellitus” (honey sweet) was added by Thomas Willis (Britain) in 1675 after rediscovering the sweetness of urine and blood of patients (first noticed by the ancient Indians) [1]. In 1776, Dobson (Britain) for the first time confirmed the presence of excess sugar in urine and blood as a cause of their sweetness. Depending on the pathogenesis, diabetes is classified as type 1 and type 2. The first widely accepted classification of diabetes mellitus was published by World Health Organization (WHO) in 1980 [2] and, in modified form, in 1985 [3]. In 1980, the WHO Expert Committee proposed two major classes of diabetes mellitus, namely: Insulin Dependent Diabetes Mellitus (IDDM) or Type 1 and Noninsulin Dependent Diabetes Mellitus (NIDDM) or Type 2 diabetes (T2D). In 1985, the WHO expert committee omitted the terms Type 1 and Type 2, but the terms IDDM and NIDDM were retained, and a class of Malnutrition-Related Diabetes Mellitus (MRDM) was introduced [3]. In both reports (1980 and 1985), other classes of diabetes were also included, for example, Impaired Glucose Tolerance (IGT) and Gestational Diabetes Mellitus (GDM) [2, 3]. These were reflected in the subsequent International Nomenclature of Disease (IND) in 1991 and in the tenth revision of the International Classification of Diseases (ICD-10) in 1992. The 1985 classification was widely accepted and used internationally even today. 

Since last few decades, diagnosis of diabetes is not only limited in blood and urine sugar levels but also in many other parameters and factors such as serum insulin levels, blood glycated haemoglobin and proteins, glucose tolerance ability, insulin sensitivity or insulin resistance, pancreatic beta-cell function, and so forth. Apart from above-mentioned parameters related abnormalities, diabetes patients are often suffered from other diabetes related complications such as—diabetic neuropathy, diabetic cardiomyopathy, diabetic nephropathy (DN), and diabetic retinopathy. These are usually caused by the poor glycemic control or improper management of diabetes mellitus. About 50% of people with diabetes are affected with one or more of the above complications. Amongst others, diabetic neuropathy is one the leading and painful complications usually suffered by many diabetic patients; however, the pathogenesis of this complication is still not fully understood due to the absence of an authentic animal model which fully mimics the complications of human diabetic neuropathy.

Animal models in diabetes research are very common when most of the existing models are developed as a conventional model either for Type 1 or for T2D. But very often a conventional model of diabetes cannot demonstrate the specific pathogenesis of diabetes related complications. Therefore, the necessity of the individual and specific model for diabetic complications has been raised in the recent years to achieve the authentic outcomes of specific research aims. A number of animal models of diabetic neuropathy have been developed in last few decades approaching from diverse point of views. However, most of them did not receive much popularity because of their considerable number of limitations and disadvantages. In a comprehensive review, Harati [4] reported that the major handicap in studying diabetic neuropathies is the lack of a suitable animal model that addresses acute and chronic events leading to diabetic neuropathy. Hence, in this review, the pathogenesis, advantages, disadvantages, and limitations of several genetic and nongenetic animal models of diabetic neuropathy have been discussed to substantiate their efficacy for human study and in order to guide diabetes research groups to more accurately select the most appropriate models to address their specific research questions.

## 2. Animal Models in Diabetic Neuropathy

Peripheral diabetic neuropathy (PDN) is a shattering complication of diabetes and leading cause of foot exclusion [5]. Clinical indications of PDN include increased vibration and thermal perception thresholds that progress to sensory loss, occurring in conjunction with degeneration of all fiber types in the peripheral nerve [6]. A proportion of patients with PDN also describe abnormal sensations such as paresthesia, allodynia, hyperalgesia, and spontaneous pain that sometimes coexist with loss of normal sensory function [7]. According to a recent review, a number of studies have investigated and described DN in mice, but it is difficult to compare these studies with each other or with human DN due to experimental differences including the animal strain, type of diabetes, method of induction, duration of diabetes, animal age, and gender [8]. Although two review articles [9, 10] on animal models of diabetic and some other neuropathies are published recently, none of them suggested the most suitable model in order to study the further pathogenesis of diabetic neuropathy and also for the pharmacological screening and development of antidiabetic or anti-neuropathic drug in their reviews. Shaikh and Somani [9] simply reviewed the behavioral, structural, functional, and molecular markers of Type 1 and Type 2 diabetic neurophaty while Höke [10] briefly reviewed the physiological changes in diabetic and some other peripheral neuropathies such as chemotherapy-induced peripheral neuropathy and human-immunodeficiency virus-associated sensory neuropathies. This review precisely discussed the progress with the animal models of diabetic neuropathy which have been developed in last few decades since early 1960s with their advantages, disadvantages, and limitations in order to assist scientists to more appropriately choose a model based on their specific research aims. Additionally, the characterization of neuropathy or advantages and limitations or disadvantages of most of the models are summarized in Table 1.

### 2.1. Models Developed during 1960s and 1970s

The nerve conduction and regenerative changes in experimental diabetes were first noticed by Eliasson during 1964-1965 [11, 12]; however, the first peripheral neuropathy in alloxan-diabetic rats was reported by Preston in 1967 [13] then Lovelace in 1968 [14]. After that a number of scientists reported diabetic neurophaty mostly in alloxan-induced diabetic models. A complete animal (rat) model of diabetic neuropathy (DN) was first reported by Jakobsen and Lundbeck in 1976 [15] with reduced sizes of nerve fiber, axon, and myelin sheath, which contribute in impaired motor function in streptozotocin (STZ)-induced diabetic rats. After a couple of years, during 1978–1980, animal model of PDN was first reported as well as evaluated by Sima and Robertson in several studies conducted in streptozotocin-induced diabetic rats and mutant diabetic [C57BL/Ks (db/db)] mice [16–18]. The PDN was initially characterized by severely decreased motor nerve conduction velocity (MNCV), absence of large myelinated fibers, and axonal atrophy in this mouse model. In the further evaluation studies, axonal changes as well as axonal dystrophy were observed in the myelinated and unmyelinated fibers followed by loss, shrinkage, and breakdown of myelin sheath in the later stage. However, the major limitation is that none of these models have been evaluated by using anti-diabetic or antineuropathic drugs.

### 2.2. Models Developed during 1980s

In early 1980s, PDN was assessed in diabetic Chinese hamster by Kennedy and colleagues [19]. Conduction velocities in both motor and sensory components of the hind lamb nerves were reduced 16–22% in diabetic compared to control animals. However, there was no reduction in nerve fiber diameters or other signs of abnormal morphology that could be correlated with these physiological effects. However, PDN in diabetic hamster is less severe than human DN in its clinical stage. Hence, further study is warranted to use this animal as a model for human PDN. Cornblath et al. [20] tried to develop a primate model of PDN in rhesus monkey. They found significantly reduced motor nerve conduction velocities and prolonged F-wave latencies in diabetic animals compared to nondiabetic control animals, while motor-evoked amplitudes did not differ. Additionally, nerve conduction times were increased in motor fibers of diabetic animals two years after the onset of diabetic hyperglycemia. Although these abnormalities are similar to those seen in humans, further study is needed to establish this primate model for human PDN since these models have not been evaluated by any antineuropathic drugs. Additionally, after comparing with diabetic and hypoglycaemic neuropathy, Sima et al. [21] reported that diabetic neuropathy is not associated with nerve cell loss but showed marked axonal atrophy involving predominantly sensory fibers. So this particular factor needs to be considered before choosing any animal model for a diabetic neuropathic study. 

### 2.3. Models Developed during 1990s

#### 2.3.1. Spontaneously Diabetic WBN/Kob Rat Model

In early 1990s, the model of PDN further developed in a spontaneously diabetic WBN/Kob rats via examining electrophysiologic, biochemical, and structural changes of peripheral nerves at 12 and 20 months of ages [26]. This model was characterized by slower motor nerve conduction and temporal dispersion of compound muscle action potential. Structural de- and remyelinations were observed in the sciatic and tibial nerves in 12-month-old rats, while 20-month-old rats additionally showed axonal degeneration and dystrophy, reduced myelinated fiber occupancy, and decreased mean myelinated fiber size. Additionally, these neuropathic manifestations are unique as compared with those found in other spontaneously diabetic animal models. This model of WBN/Kob rats is further supported by Ozaki et al. [38], because this model of PDN develops primary segmental demyelination and secondary axonal degeneration, which are similar to those in human patients with diabetes mellitus and unlike those in rodents with streptozotocin-induced diabetes [38]. Hence, spontaneously diabetic WBN/Kob rats can be a better model to study the human PDN. 

#### 2.3.2. L-Fucose-Induced Rat Model

In late 1990s, it has been reported that L-fucose, a competitive inhibitor of sodium-dependent myoinositol transport, has been shown effective to induce diabetic neuropathy in normal rats mediated by Na^+^-K^+^-ATPase activity and conduction of nerve velocity [27]. To further validate, long-term feeding of L-fucose has been studied in this model and evaluated by nerve Na^+^-K^+^-ATPase activity, conduction velocity, and myelinated nerve fiber pathology. After 24-week supplementation of L-fucose enriched (10 or 20%) diets, Na^+^-K^+^-ATPase activity was significantly decreased, associated with a 25–30% reduction in nerve conduction velocity. Twenty percent L-fucose diet resulted in significant axonal atrophy, paranodal swelling, and paranodal demyelination without increasing Walleran degeneration or nerve fiber loss. After this study, it has been recommended that this L-fucose model can serve as an experimental tool to study the diabetic neuropathy.

#### 2.3.3. Partial Sciatic-Nerve Ligated Rat Model

In another study, partial ligation of sciatic nerve method has been used to induce PDN and compared with a usual STZ-induced rat model of PDN [28]. STZ-induced diabetic animals were chronically ill, with reduced growth rate, polyuria, diarrhoea, and enlarged and distended bladders when these symptoms were not found in sciatic nerve ligated model. This sciatic nerve ligated model has also been evaluated with antineuropathic drugs (Morphine and L-Baclofen), which produce greater reversal of mechanical hyperalgesia following partial nerve ligation. They also added that STZ-induced diabetes in rats produces long-lasting mechanical but not thermal hyperalgesia. Although evaluated by antineuropathic drugs, further study is needed to understand the induction of the major pathogenesis of PDN.

### 2.4. Models Developed during 2000s

#### 2.4.1. Nonobese Diabetic (NOD) Mice Models

Diabetic autonomic neuropathy has been examined in the nonobese diabetic (NOD), and streptozotocin (STZ)-induced diabetic mice, two models of Type 1 diabetes, and the db/db mouse, a model of Type 2 diabetes [29]. It was found that after only 3–5 weeks of diabetes, NOD mice developed markedly swollen axons and dendrites (neurotic dystrophy) in the prevertebral superior mesenteric and celiac ganglia (SMG-CG), similar to the pathology described in diabetic STZ- and BBW-rat and human. STZ-induced diabetic mice develop identical changes, although at a much slower pace and to a lesser degree than NOD mice. Chronically diabetic Type 2 db/db mice fail to develop neurotic dystrophy, suggesting that hyperglycaemia alone may not be the crucial and sufficient element. Therefore, NOD mouse appears to be a valuable model of diabetic sympathetic autonomic neuropathy which is consistent with the pathogenesis of other rodent models and human. It has been further supported by a very recently published comparative study on peripheral neuropathy between NOD and ICR diabetic mice [30] where NOD mice have been suggested as a better model than ICR mice particularly in terms of nerve regeneration. 

#### 2.4.2. Genetic Rodent Models

The development of peripheral diabetic neuropathy has been assessed by longitudinal memory performance in spontaneously induced Type 1 diabetic Ins2C96Y Akita mice by Choeiri et al. [31]. This model was characterized by reduced number of beta cells with hypoinsulinemia, progressive hyperglycemia, and reduced sensory nerve conduction velocity; however no significant deficit has been detected as Morris water maze trial compared to the control group, and many other diabetic neuropathy-related major parameters have not been measured. Later, after measuring a number of diabetic neuropathy related parameters, Schmidt et al. [32] reported that Ins2 Akita mouse is a robust model of diabetic sympathetic autonomic neuropathy which closely corresponds to the characteristics pathology of other rodent models and humans. This model has been evaluated by progressively developed markedly swollen axons and dendrites which are the common signs of neurotic dystrophy. According to the above-mentioned studies, although Ins2 Akita mice can be a proper genetic model of diabetic neuropathy, this model needs to be evaluated by antidiabetic and antineuropathic drugs. 

Drel et al. [6] reported that leptin-deficient (ob/ob) mice clearly manifest thermal hypoalgesia, the condition observed in human subjects, which is a transient phenomenon in PDN in humans [39] and, non-fasting blood glucose was not more than 20 mmol/L which was found very higher, ~30 mmol/L, in Zucker Diabetic Fatty (ZDF) rats [39]. The ob/ob mice developed a clearly manifested slow motor and sensory nerve conduction and accumulation of peripheral nerve sorbitol pathway intermediate when fed a regular mouse diet to maintain moderated hyperglycaemia [6]. Usually subject with Type 1 or Type 2 diabetes display epidermal nerve fiber loss, and it was found that 11-week-old ob/ob mice developed a dramatic reduction (78%) in intraepidermal nerve fiber compared with age-matched nondiabetic controls [6]. This animal model was also successfully evaluated by a potent inhibitor of PDN such as aldose reductase inhibitor which normalized motor and sensory nerve conduction velocity. The results of this study suggest that leptin-deficient ob/ob mice can be better for PDN.

On the other hand, Kamenov et al. [33] compared the complications of diabetic neuropathy between Otsuka Long-Evans Tokushima Fatty (OLETF) rats and Long-Evans Tokushima Otsuka (LETO) rats, where OLETF is a spontaneous animal model of T2D. In this regard, each type of animal has been divided into 2 subgroups and fed with or without sucrose-containing diets for 2 months and found that the blood glucose and HbA1c levels were significantly higher in OLETF rats, when compared with those in control LETO rats. Motor nerve conduction velocity and thermal nociception were significantly decreased in OLETF rats in their 10 months of age, while the values of the tail pressure test did not differ compared with those from LETO rats. It was concluded that signs of diabetic neuropathy appear in LETO rats after a longer period of time compared to OLETF rats. Therefore OLETF rat can be a better animal model for Type 2 diabetic neuropathy than the LETO rats.

Recently, Serafín et al. [34] developed a model of diabetic neuropathy in 6-week-old rat insulin I promoter/human interferon-beta (RIP/IFN*β*) transgenic ICR mice with a low dose of STZ injection (30 mg/kg BW) for 5 consecutive days. Additionally, in order to induce nerve damage, after 4 weeks of sustained hyperglycemia, the left sciatic nerve was exposed by blunt dissection and crushed at the femur major trochanter level for three times in succession for 30 seconds in anaesthetized animals when intact contralateral nerve was used as a control. This transgenic model was evaluated by significant hyperglycemia, slower tibial sensory nerve conduction velocity (SNCV) and increased motor latencies and duration of compound muscle potential, reduced nerve fiber density, and so on. The slower recovery of nerve conduction velocities were observed in the diabetic transgenic mice group compared to the control. Although this model has been displayed most of the major pathogenesis of peripheral diabetic neuropathy, a sophisticated surgical approach has been used with multiple STZ injections to develop this model, and it has not been evaluated by any antidiabetic or antineuropathic drugs. 

#### 2.4.3. Experimentally-Induced Models

Filho and Fazan [22] developed a streptozotocin (STZ)-induced model of phrenic nerve neuropathy in rats. Diabetes was induced by a single injection of streptozotocin to penile vein, and higher blood glucose level confirmed the diabetic state. Left and right fascicular areas and diameter of the phrenic nerves were significantly decreased in the proximal segments and right segments, respectively. The phrenic nerves of diabetic rats showed smaller myelinated axon diameters compared to controls. The *g* ratio for diabetic rats was significantly lower than the controls when these changes have been restored by the daily injection (s.c.) of insulin (9 U/kg body weight). Although this model has been evaluated by insulin, no antineuropathic drug has been used for the evaluation of this model.

After a year, Obrosova and colleagues [35] tried to develop a neuropathy model in female C57BL6/J mice by feeding high-fat diet for a 16-week period. This model was characterized by the deficit of motor and sensory nerve conductions, tactile allodynia, and thermal hypoalgesia; however intradermal nerve fiber loss or axonal atrophy was absent in this model. Although plasma FFA and insulin concentrations were increased and glucose tolerance was impaired, the frank hyperglycemia was absent in this model. According to the data, although this model can be used for prediabetes and obesity related neuropathy, it cannot be used for chronic diabetic neuropathy. This model has also not been evaluated by any antineuropathic drug, and the duration of model development time is one of the major concerns.

In 2008, Hong and Kang [40] published a very special finding on auditory neuropathy in streptozotocin-induced diabetic ICR mice in order to understand the possible auditory damage. The diabetes was induced by the different dosages of STZ (50, 100, and 150 mg/kg BW) dissolved in citrate buffer (pH 4.5) in 7-week-old male animals. The auditory diabetic neuropathy in this particular model has been evaluated by significantly increased absolute latencies of IV, and the interpeak latencies of I–III and I–IV of auditory brainstem response (ABR), and dose dependent induction of Pa latency of auditory middle latency response (AMLR) in STZ treated mice compared to control mice. In terms of ABR, best results were observed for the dose of 100 mg/kg BW of STZ compared to other two STZ dosages. From the data of this study, authors suggested that the STZ-induced mouse can be used for the evaluation of auditory pathway impairment via ABR and AMLR tests, however this model has not been evaluated by any antidiabetic or antineuropathic drugs.

At the same year, Vareniuk et al. [24] compared the pathogenesis of peripheral diabetic neuropathy in STZ-induced wild-type and inducible nitric oxide synthase (iNOS) gene deficient mice with C57BL6/J background. The model was developed by injecting single doses (100 mg/kg BW) of STZ injection (i.p.) to nonfasted wild-type and iNOS (also known as Nos2) deficient (iNos (−/−)) mice and maintained for a 6-week experimental period. Although STZ-injected wild-type mice displayed peroxynitrite injury in peripheral nerve and dorsal root ganglion neurons and developed motor and sensory nerve conduction velocity deficits, thermal and mechanical hypoalgesia, tactile allodynia, and approximately 36% loss of intraepidermal nerve fibers, the STZ-injected iNOS (−/−) mice did not display most of the above-mentioned pathogenesis except nitrosative stress in dorsal root ganglia with normal nerve conduction velocities and less severe small fiber sensory neuropathy. Although the STZ injected model was not evaluated by any antidiabetic or antineuropathic drugs, but from this study it is clear that iNOS gene plays a major role in the induction or peripheral diabetic neuropathy which can be future research and drug development target.

Recently, Muthuraman and colleagues [36] developed a rat model of vasculatic neuropathy by ischemic perfusion in the rat femoral artery. This model was validated after 2, 4, and 6 h of ischemia followed by prolonged reperfusion. The model has been characterized by thermal and mechanical hyperalgesia in paw and tail which are associated with peripheral and central neuropathic pain, respectively. The serum IL-10, nerve fiber density, and nerve conduction velocity were lower, and serum nitrate, malondialdehyde (MDA) and TNF-alpha levels were higher in this model. Although neuropathy induction period of this model is very short and has similar pathogenesis with human diabetic neuropathy, the pathogenesis of neuropathy have not been developed here via hyperglycaemia, what is usually happened in diabetic neuropathy, but via ischemic perfusion in the animal femoral artery. Hence, this model cannot be a better model to study human peripheral diabetic neuropathy. Additionally, this model has not been evaluated by using any antineuropathic drugs.

### 2.5. Models Developed during 2010s

#### 2.5.1. Genetically Modified SDT Rat Model

Recently, Yamaguchi et al. [37] developed diabetic peripheral neuropathy in Spontaneously Diabetic Torii (SDT) fatty rats by introducing *fa* allele of Zucker Diabetic Fatty (ZDF) rats since SDT rats develop delayed hyperglycemia compared to diabetic complications. Apart from common diabetic abnormalities such as sustained hyperglycemia and dyslipidemia, this diabetic peripheral neuropathic model was further characterized by significantly delayed and lower motor nerve conduction velocity from 24 weeks and significantly lower number of sural nerve fibers at the end of the 40-week experimental period. Additionally, thickened epineurial arterioles were frequently found in this model. This model was further evaluated by an antidiabetic drug such as pioglitazone which could significantly improve the motor nerve conduction velocity and blood HbA1c level when fed food admixture at a dose of 10 mg/kg/day for a 6-week period. So this model can be a better diabetic peripheral neuropathic model not only to understand the pathogenesis of diabetic peripheral neuropathy but also to screen and develop antidiabetic peripheral neuropathic drug, particularly for Type 2 diabetes.

#### 2.5.2. Genetically Modified C57BLKS Mice Model

Very recently, Hinder et al. [23] developed a dyslipidemia-induced mouse model of diabetic neuropathy by some genetic manipulation. This model was developed by knockout of ApoE and ApoB48 genes in db/db or ob/ob mice C57BLKS background which mimicked the neuropathic plasma lipid profile in diabetic humans. It was also characterized by increased body weight, hyperlipidemia, hyperglycemia, and the evidence of neuropathy; however this model was not delivered by lipid profile usually seen in translational diabetic neuropathy. Although this model has been characterized by significantly lower tail flick response to heat stimulus, sciatic motor nerve conduction velocity, and intraepididymal nerve fiber velocity, mismatched results were observed for the body weight, blood glucose, plasma lipids, and total blood glycated haemoglobin. From the results of this study, authors suggested that the overall effects of ApoE knockout, either directly upon nerve structure and function or indirectly on lipid metabolism, are insufficient to significantly alter the course of translational diabetic neuropathy research, and further therapeutic intervention is necessary in this regard. Apart from the above limitations, this model was also not evaluated by any antidiabetic or antineuropathic drug.

#### 2.5.3. Streptozotocin-Induced Diabetic Sensory Neuropathy Mice Model

Most recently, Murakami et al. [25] developed a sensory neuropathy model in STZ-induced 8-week-old ddY mice. Diabetes was developed by a single injection (i.p.) of STZ and confirmed by blood glucose level >16.7 mmol/L one week after the STZ injection. This model has been evaluated by significantly lower sensory nerve conduction velocity (SNCV), higher nociceptive threshold, hypoalgesia, and reduced axon area of unmyelinated nerve fibers or unmyelinated fiber atrophy. Although no difference was found for the myelinated nerve fiber areas between the diabetic and healthy mice, this model has been successfully evaluated by insulin treatment. Since the unmyelinated nerve fibers were more affected than myelinated nerve fibers and it has been successfully evaluated with insulin treatment, so it can be a better model to study the human sensory polyneuropathy. 

## 3. Conclusion

As per this review, although a number of approaches have been used to develop the diabetic neuropathic models in different strains of animals in last five decades, none of them are without limitations. Several models such as conventional and genetically modified C57BL/Ks (db/db) mice, streptozotocin-induced C57BL6/J and ddY mice, spontaneously diabetic WBN/Kob rats, L-fucose induced neuropathic rats, nonobese diabetic (NOD) rats, spontaneously induced InS2 Akita mice, leptin-deficient (ob/ob) mice, high-fat diet-fed female C57BL6/J mice, and genetically modified SDT fatty rats have been shown to develop major pathogenesis of diabetic neuropathy or peripheral diabetic neuropathy; however most of them were not validated either by antidiabetic or antineuropathic drugs. Some models such as streptozotocin-induced rats, Chinese hamster, rhesus monkey, partial sciatic nerve ligated rats, and Otsuka Long-Evans Tokushima Fatty (OLETF) rats developed very few major or some minor pathogenesis of diabetic neuropathy and peripheral diabetic neuropathy and the model development time for some of these models were very long. The best model of diabetic neuropathy or peripheral diabetic neuropathy should have some major criteria such as: (1) the model should have all major pathogenesis of diabetic neuropathy or PDN with other minor pathogenesis which is normally found in human diabetic neuropathic patients, (2) the model should be sensitive to antidiabetic or anti-neuropathic drugs, and (3) the model needs to be suitable to study the pathogenesis of disease as well as for routine pharmacological screening of antidiabetic anti-neuropathic drugs. Although most of the genetic or genetically modified models of diabetic neuropathy or PDN discussed in this review are suitable for studying the pathogenesis of the diseases, the C57BL/Ks (db/db) mice, streptozotocin-induced C57BL6/J and ddY mice, spontaneously diabetic WBN/Kob rats, nonobese diabetic mice, spontaneously induced Ins2 Akita mice, and leptin-deficient (ob/ob) mice have been found as better models for human diabetic neuropathy when high-fat diet-fed female C57BL6/J mice have been suggested to use for prediabetic or obesity related diabetic neuropathy. Although L-fucose induced neuropathic rats, OLETF rats, and genetically modified SDT rats have shown some promising pathogenesis of diabetic and PDN, further studies are needed to understand the suitability and usefulness of these models for diabetic or peripheral diabetic neuropathic researches.

## Figures and Tables

**Table 1:** Characterization criteria (advantages) and limitations (disadvantages) of some selective animal models of diabetic neuropathy developed since 1960s.

Animals models	References	Characterization of diabetic neuropathy/advantages	Limitations/disadvantages
Streptozotocin-induced rat model (classic)	Jakobsen and Lundbeck [15].	(i) Reduced sizes of nerve fiber, axon, and myelin sheath. (ii) Impaired motor function.	Not validated by antineuropathic drug.

Streptozotocin-induced rat model (recent)	Filho and Fazan [22].	(i) Significantly reduced right and left fascicular areas and myelination of phrenic nerves. (ii) Validated by insulin (s.c.).	(i) Some major pathogenesis of diabetic neuropathy has not been characterized. (ii) Although validated by insulin (s.c.), no antineuropathic drug has been used.

C57BL/Ks (db/db) mice model (classic)	Sima and Robertson [16, 17]; Robertson and Sima [18].	(i) Severely decreased motor nerve conduction velocity (MNCV). (ii) Absence of large myelinated fibers. (iii) Axonal atrophy. (iv) Axonal dystrophy in myelinated and unmyelinated fibers. (v) Loss, shrinkage, and breakdown of myeline sheath.	Not evaluated by any anti-diabetic or antineuropathic drug.

Genetically modified C57BL/Ks (db/db) mice model (recent)	Hinder et al. [23].	(i) Increased body weight, hyperglycemia, and hyperlipidemia. (ii) Lower tail flick response to heat stimulus, sciatic motor nerve conduction velocity, and intraepididymal nerve fiber velocity.	(i) Mismatched results were observed for body weight, blood glucose, plasma lipids, and blood glycated hemoglobin. (ii) Not validated by anti-diabetic or antineuropathic drugs.

Streptozotocin-induced C57BL6/J mice model	Vareniuk et al. [24].	(i) Peroxynitrite injury in peripheral nerve and dorsal root ganglion neurons. (ii) Motor and sensory nerve conduction velocity deficits, thermal and mechanical hyperplasia, tactile allodynia, and loss of intraepidermal nerve fibers.	Not validated by using antineuropathic drug.

Streptozotocin-induced diabetic sensory neuropathic ddY mice model	Murakami et al. [25].	(i) Significantly lower sensory nerve conduction velocity, higher nociceptive threshold, hypoalgesia, and unmyelinated fiber atrophy. (ii) Successfully evaluated by insulin treatment. (iii) Can be a better model to study the human sensory polyneuropathy.	No significant change was found in the myelinated nerve fiber areas.

Chinese hamster neuropathic model	Kennedy et al. [19].	Reduced conduction velocity of both motor and sensory components of hind lamb nerves (16–22%).	(i) Peripheral diabetic neuropathy (PDN) was less severe than human diabetic neuropathy. (ii) Further study needed for proper validation.

Rhesus monkey model of PDN	Cornblath et al. [20].	(i) Significantly reduced motor conduction velocity. (ii) Prolonged F-wave latencies. (iii) Pathogeneses' resembles to humans.	(i) No difference in motor-evoked amplitudes. (ii) Prolonged nerve conduction induction time (2 years). (iii) Not validated by antineuropathic drug.

Spontaneously diabetic WBN/Kob rat model	Yagihashi et al. [26].	(i) Slower motor nerve conduction and temporal dispersion of compound muscle action potential. (ii) Structural de- and remyelination in the sciatic and tibial nerves at 12 month. (iii) Axonal degeneration, dystrophy, and reduced myelinated fiber at 20 month. (iv) Resembles human pathogenesis of PDN.	Not validated by antineuropathic drug.

L-fucose induced neuropathic rat model	Sima et al. [27].	(i) Reduced Na^+^-K^+^-ATPase activity. (ii) Reduced nerve conduction velocity. (iii) Axonal dystrophy. (iv) Paranodal swelling and demyelination without increasing Walleran degeneration of nerve fiber loss.	Not validated by antineuropathic drug.

Partial sciatic nerve ligated rat model	Fox et al. [28].	(i) Produced long-lasting mechanical, but thermal hyperalgesia. (ii) Evaluated by ant-diabetic neuropathic drugs.	Major pathogenesis was not characterized.

Nonobese diabetic (NOD) mice model	Schmidt et al. [29]; Homs et al. [30].	(i) Short induction period. (ii) Markedly swollen axons and dendrites (neurotic dystrophy). (iii) Consistent with the pathogenesis of other rodent models of PDN and human PDN. (iv) Suggested as a better model than ICR mice particularly in terms of nerve regeneration.	Not validated by antineuropathic drug.

Spontaneously induced Ins2 Akita mouse model	Choeiri et al. [31]; Schmidt et al. [32].	(i) Spontaneously induced diabetic model. (ii) Progressive and sustained chronic hyperglycemia. (iii) Reduced sensory nerve conduction velocity. (iv) Markedly swollen axons and dendrites (neurotic dystrophy). (v) Consistent with the pathogenesis of other rodent models of PDN and human PDN.	Not validated by anti-diabetic or antineuropathic drug.

Leptin-deficient (ob/ob) mice model	Drel et al. [6].	(i) Clearly manifested thermal hypoalgesia. (ii) Relatively higher nonfasting blood glucose level (20 mmol/L). (iii) Slow motor and sensory nerve conduction. (iv) Significant reduction of intraepidermal nerve fiber. (v) Validated by antiperipheral diabetic neuropathic drug.	May not be widely available for routine pharmacological screening of anti-diabetic or anti-neuropathic drugs.

Otsuka Long-Evans Tokushima Fatty (OLETF) rats model	Kamenov et al. [33].	(i) Significantly higher blood glucose and HbA1c levels. (ii) Reduced motor nerve conduction velocity and thermal nociception.	(i) Some major pathogenesis of PDN has not been characterized. (ii) Not validated by anti-diabetic neuropathic drugs.

Rat insulin I promoter/human interferon-beta (RIP/IFN*β*) transgenic ICR mice model	Serafín et al. [34].	(i) Significantly hyperglycemia, slower tibial sensory nerve conduction velocity. (ii) Reduced nerve fiber density and increased motor latencies.	(i) A sophisticated surgical approach has been used to develop the model. (ii) Not validated by anti-diabetic or antineuropathic drugs.

High-fat diet-fed female C57BL6/J mice model	Obrosova et al. [35].	(i) Deficit of motor and sensory nerve conductions, tactile allodynia, and thermal hypoalgesia. (ii) Can be used as model for prediabetic or obesity related neuropathy.	(i) Intradermal nerve fiber loss, and axonal atrophy was absent. (ii) Cannot be used for chronic diabetic neuropathy. (iii) Not validated by antineuropathic drugs.

Surgically-induced neuropathic model	Muthuraman et al. [36].	(i) Thermal and mechanical hyperalgesia in paw and tail. (ii) Reduced nerve fiber density and nerve conduction velocity. (iii) Very short induction period.	(i) Not validated by using antineuropathic drug. (ii) Not suitable to study the human diabetic neuropathy.

Genetically modified SDT fatty rat model	Yamaguchi et al. [37].	(i) Sustained hyperglycemia and dyslipidemia with delayed and reduced motor nerve conduction velocity. (ii) Lower number of sural nerve fibers and thickened epinural arterioles. (iii) Successfully validated by anti-diabetic drug such as pioglitazone.	Some pathogenesis was induced only after a long period of time such as 40 weeks.

## List of Abbreviations (in Alphabetical Order)

DN: Diabetic neuropathy
GDM: Gestational diabetes mellitus
ICD: International classification of diseases
IDDM: Insulin dependent diabetes mellitus
IFN: Interferon
IGT: Impaired glucose tolerance
IND: International nomenclature of diseases
iNOS: Inducible nitric oxide synthase
LETO: Long Evans Tokushima obese
MNCV: Motor nerve conduction velocity
MRDM: Malnutrition related diabetes mellitus
NIDDM: Noninsulin dependent diabetes mellitus
NOD: Nonobese diabetic
OLETF: Otsuka long Evans Tokushima fatty
PDN: Peripheral diabetic neuropathy
SDT: Spontaneously diabetic torii
SNCV: Sensory nerve conduction velocity
STZ: Streptozotocin
T2D: Type 2 diabetes
WHO: World health organization
ZDF: Zucker diabetic fatty.
